# Comprehensive Gene-Expression Survey Identifies Wif1 as a Modulator of Cardiomyocyte Differentiation

**DOI:** 10.1371/journal.pone.0015504

**Published:** 2010-12-13

**Authors:** Henk P. J. Buermans, Bram van Wijk, Margriet A. Hulsker, Niels C. H. Smit, Johan T. den Dunnen, Gertjan B. van Ommen, Antoon F. Moorman, Maurice J. van den Hoff, Peter A. C. 't Hoen

**Affiliations:** 1 Human and Clinical Genetics/Leiden University Medical Center, Leiden, The Netherlands; 2 Heart Failure Research Center/Academic Medical Center, Amsterdam, The Netherlands; Sanford-Burnham Medical Research Institute, United States of America

## Abstract

During chicken cardiac development the proepicardium (PE) forms the epicardium (Epi), which contributes to several non-myocardial lineages within the heart. In contrast to Epi-explant cultures, PE explants can differentiate into a cardiomyocyte phenotype. By temporal microarray expression profiles of PE-explant cultures and maturing Epi cells, we identified genes specifically associated with differentiation towards either of these lineages and genes that are associated with the Epi-lineage restriction. We found a central role for Wnt signaling in the determination of the different cell lineages. Immunofluorescent staining after recombinant-protein incubation in PE-explant cultures indicated that the early upregulated Wnt inhibitory factor-1 (Wif1), stimulates cardiomyocyte differentiation in a similar manner as Wnt stimulation. Concordingly, in the mouse pluripotent embryogenic carcinoma cell line p19cl6, early and late Wif1 exposure enhances and attenuates differentiation, respectively. In ovo exposure of the HH12 chicken embryonic heart to Wif1 increases the Tbx18-positive cardiac progenitor pool. These data indicate that Wif1 enhances cardiomyogenesis.

## Introduction

The mammalian heart has a limited regenerative capacity. As a direct consequence, restoration of proper cardiac function following cardiomyocyte loss, due to myocardial infarction or ischaemic heart disease, is severely impaired. Many research projects have explored the means to re-populate damaged cardiac tissue with functional cardiomyocytes using stem cells from either endogenous or exogenous sources which have the potential to differentiate into cardiomyocytes. Various signal transduction pathways have been implicated in cardiogenesis like the Tgf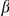 [1] and Wnt [2] cascades. However, crosstalk between signal transduction pathways is extensive and the exact mechanism by which precursor cells differentiate into the myocardial lineage remains largely unknown.

Several groups have used temporal microarray gene-expression profiling using in vitro pluripotent cell models in order to identify genes and processes associated with cardiomyocyte differentiation. Peng et al. [3] were the first to use cDNA microarrays to study the mouse pluripotent embryogenic carcinoma cell line p19cl6 differentiating towards cardiomyocytes, while more recently Beqqali et al. [4] determined time-dependent gene-expression profiles during human embryonic stem cells differentiation. Although multiple technical and biological differences exist between these two studies, a literature-aided meta-analysis [5] on these data, indicated several common biological processes and functional concepts to be associated with the observed differentially-expressed genes in these two studies. The top contributing concepts being Wnt signaling (Lef1, Axin2, Vangl1, Wnt3A, Dkk1) and cardiac transcription factors (Gata4, Hand1, Mef2c, Lhx1) (Buermans et al., unpublished results), demonstrating cardiogenic differentiation is largely conserved across species.

The proepicardium (PE) is a second heart field derived, villous non-myocardial outgrowth protruding into the pericardial cavity adjacent to the inflow tract. During subsequent embryonic development, the PE attaches to and covers the embryonic heart tube, giving rise to the embryonic Epicardium (Epi). The Epi in turn contributes precursors for several non-myocardial lineages within the heart including coronary smooth muscle cells, coronary endothelium and cardiac fibroblasts. Spontaneous myocardial differentiation in chicken PE-explant cultures was first described by Langford et al. [6]. More recent studies yielded more definite insights into the processes involved [7]–[9]. The formation of the PE from the pericardial mesoderm is regulated by a delicate spatial distribution of members of the Bmp and Fgf growth factor family [9]. Although epicardial lineage analysis have suggested a small myocardial contribution of epicardial origin, cultured epicardial cells do not differentiate into myocardial cells. Cultured proepicardial cells, in contrast, spontaneously differentiate into myocardial cells. Thus, in the short period of time between the emergence of the PE and the subsequent formation of the Epi, these cells loose the potential to differentiate towards the cardiomyocyte lineage. This implies major changes in the gene-expression profile that restricts the myocardial differentiation potential upon attachment of the PE to the embryonic myocardium. We refer to these changes as the “epicardial lock”. The Epi is maintained in this state in the adult heart. The PE and its derived cell types are of particular interest for adult cardiac regeneration due to their innate ability to contribute to all major cardiovascular lineages. Identifying genes and processes that underlie the “epicardial lock” may provide insight towards cardiac regeneration therapies in which epicardial and/or epicardial derived cells are reprogrammed such that the myocardial differentiation potential is reactivated.

Chicken have been used as a model for cardiac developmental biology for many years mainly due to the fact that the embryos can be manipulated in ovo, the heart initially develops outside the pericardial cavity, cardiac development can be precisely timed [10], and tissue and organ size is overall larger than for their mouse or rat counterparts. With the recent release of the WASCHUC 2006 genome and the development of chicken oligonucleotide microarrays, genome-wide gene-expression analyses have become feasible. In the present study we determined gene-expression profiles in PE-explant cultures during cardiomyocyte differentiation as well as in various stages of epicardial maturation using chicken oligonucleotide microarrays representing 20460 transcripts. We identified several groups of co-regulated genes associated with different stages during cardiomyocyte differentiation. We also show that the early stages during Epi cell maturation are associated with a relatively low number of differentially-expressed genes. Furthermore, by integrating these two data sets we assessed divergent gene-expression profiles between the myocardial and Epi lineages and found a central role for Wnt signaling to be associated with the “epicardial lock”. We next performed a series of functional interventions in chicken embryos, PE-explant cultures and mouse p19cl6 cells to show that Wnt inhibitory factor-1 enhances cardiomyocyte differentiation by increasing the cardiac progenitor pool.

## Results

### Dynamic changes in gene expression during proepicardial to cardiomyocyte differentiation

**Figure 1:**
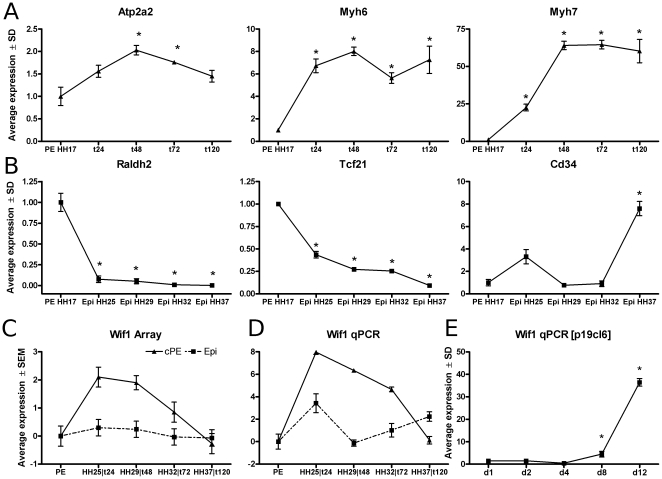
PE-Epi explant qPCR. Expression levels for hallmark genes during PE (A; Atp2a2, Myh6 & Myh7) and Epi (B; Raldh2, Tcf21 & Cd34) cultures. Correction was applied to the relative expression values in order to remove multiplicative between-session variation [52]. Stage HH16 PE expression has been set to 1. Bars represent mean expression levels 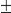 SD. * indicates a significant difference in gene expression relative to HH16 PE. Confirmation of microarray gene-expression profiles for Wif1 (C&D) with qPCR. Y-axis represents log2-transformed mean-expression levels 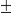 SEM. Stage HH16 PE expression has been set to 0. PE-explant cultures at 24, 48, 72 and 120 hours were compared with all four Epi stages. NADH dehydrogenase (ubiquinone) Fe-S protein 3, 30kDa (NADH-coenzyme Q reductase)(Ndufb3) was used as an internal control to normalize gene expression. Gene expression profiles for Wif1 (E) during p19cl6 differentiation towards a cardiac-myocyte phenotype. Hypoxanthine-guanine phosphoribosyltransferase (Hprt) was used as an internal control to normalize qPCR gene-expression levels. Lines represent mean gene-expression levels 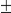 SD calculated relative to time matched controls. * indicates a significant difference in gene expression relative to control conditions.

PE were explanted on collagen gels and cultured for up to five days to allow differentiation into the cardiomyocyte lineage. qPCR analysis showed that the expression of the myocardial markers Atp2a2, Myh6 and Myh7 reached maximum levels at 48 hours (Figure 1A). From 72 hours onward spontaneously-beating cardiomyocytes were observed. These data confirmed successful in vitro cardiomyocyte differentiation from chicken PE cells.

**Figure 2:**
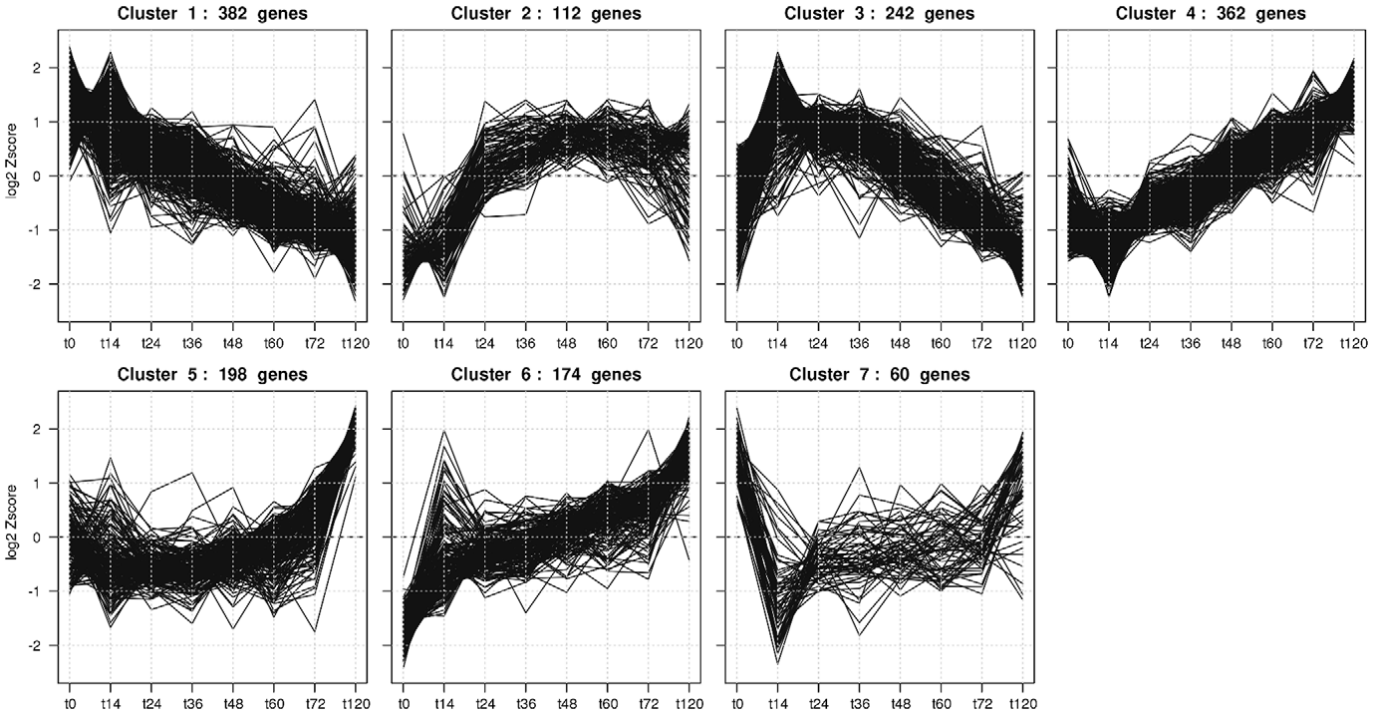
Kmeans clusters. Kmean clustering of significantly differentially-expressed genes during PE-explant cardiomyogenesis. Zscore transformed expression levels were clustered on Pearson correlation.

Gene-expression profiles were determined prior to explanting and after 14, 24, 36, 48, 60, 72 and 120 hours in culture. We chose a 2-color dye-swapped looped experiment design (Figure S1) which allows for a more accurate comparison between samples than the common-reference approach does when applied to time-course experiments [11]. Moreover, we applied the temporal Hotelling T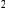-test [12], [13] to robustly detect small but consistent changes in temporal gene expression. This analysis indicated 1530 probes to be differentially expressed in time. K-means clustering discerned distinct groups of genes with correlated profiles (Figure 2). Many microarray studies subsequently rely on Gene Ontology (GO) annotation [14], i.e., relationships between genes and processes, to identify perturbed processes between experimental conditions at a higher information level. Although GO-annotation data for the chicken genome has improved considerably over the years, it is still behind on Human, Mouse or Rat. We found that the GO-annotation performs poorly when applied to chicken specific or chicken-to-human ortholog GO annotation. Moreover, the GO-annotation is potentially lagging behind on the current literature knowledge and has limited coverage of cardiac related processes and pathways. Instead, we used the Anni-v2 tool [15] to identify groups of genes with common biological functions. This tool is based on concept profiles, that summarize the literature context in which the gene is mentioned, and are compiled directly from the Pubmed records. The system is completely transparent, allowing the user to trace back the actual Pubmed records the inferred associations are based upon.

Genes in cluster 1 of the k-means clustering (Figure 2) were progressively downregulated over time. Common concepts associated with the genes in this cluster included cell-cycle progression [AurkA, Cdc2, Cdc16, Ccnb2] and DNA maintenance [Msh2, Exo1]. In contrast, genes in clusters 4 and 6 showed progressively increased expression. As expected genes in these two clusters were mainly basal cardiogenic factors implicated in myocardial differentiation. Concept analysis identified genes involved in extracellular collagen composition and maintenance [Col1A2, Col3A1 and Col6A2 in cluster 4 and Col2A1, Sparc, Tgfb3, Mmp23B and Mmp2 in cluster 6]. Also alterations in proteoglycan composition [Lum & Dcn in cluster 4], Tgf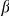 [Tgf
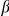
3 and Tgf
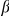
R1 in cluster 4] and Ras signaling [Rab9, Rab6a and Rab3gap2 in cluster 6] were indicated.

A distinction between early (cluster 3 & 7), intermediate (cluster 2) and late (cluster 5) changes in expression could be discerned representing cardiac specification, maintenance and maturation, respectively. Cluster 3 shows a transient increase in expression up to 24–48 hours in culture that coincides with the specification phase that precedes commitment to the cardiomyocyte lineage. This cluster contains Bmp2, a known factor involved in cardiac induction and specification [1]. Genes with correlated expression profiles are speculated to have related biological properties. Therefore, Wif1 and Fgf12, also in this cluster, represent candidate genes for cardiomyocyte specification. Cluster 2 contains genes associated with the dystrophin-glycoprotein complex and myosin light chains [Sgcb, Tpm1, Myl1,2,3, MylK] and suppressor of cytokine signaling proteins [Lifr, Cish, Socs1]. Several Wnt-signaling related genes can be found in cluster 5 which display a sharp increase in expression at day 5, e.g., two members of the secreted Wnt antagonist family, Dkk3 and Frzb, and two members of the Frizzled related receptor family, Fzd2 and Fzd7. Given the large number of differentially-expressed genes identified in this paper an in depth description of all genes in the individual clusters is not possible. A list with all differentially expressed genes is available in the Table S1.

Taken together, these data indicate that different phases during cardiomyocyte differentiation from chicken PE cells can be distinguished. Moreover, the differentially expressed genes in cluster 3 may represent previously unknown modulators for cardiac specification.

### Divergent expression profiles between PE and Epi differentiation

In contrast to PE explants, explanted Epi cells cannot differentiate into a cardiomyocyte phenotype. In order to gain more insight into the processes underlying this Epi-to-myocardial-lock, we compared the PE explant expression data with gene expression profiles derived from a series of different stages of epicardial development, i.e., prior to vessel formation (HH25), when intra-cardiac vessels have started to form (HH29), when the coronary circulation has matured but is not yet perfused (HH32) and when coronary circulation is functional (HH37). In line with previous reports, expression levels of Aldh1a2 [16] and Tcf21 [17], determined by qPCR, significantly decreased as maturation progressed, while the endothelial progenitor marker Cd34 was significantly increased at stage HH37 (Figure 1B). This indicates that our samples represent the native development of embryonic towards adult epicardium [18].

**Figure 3:**
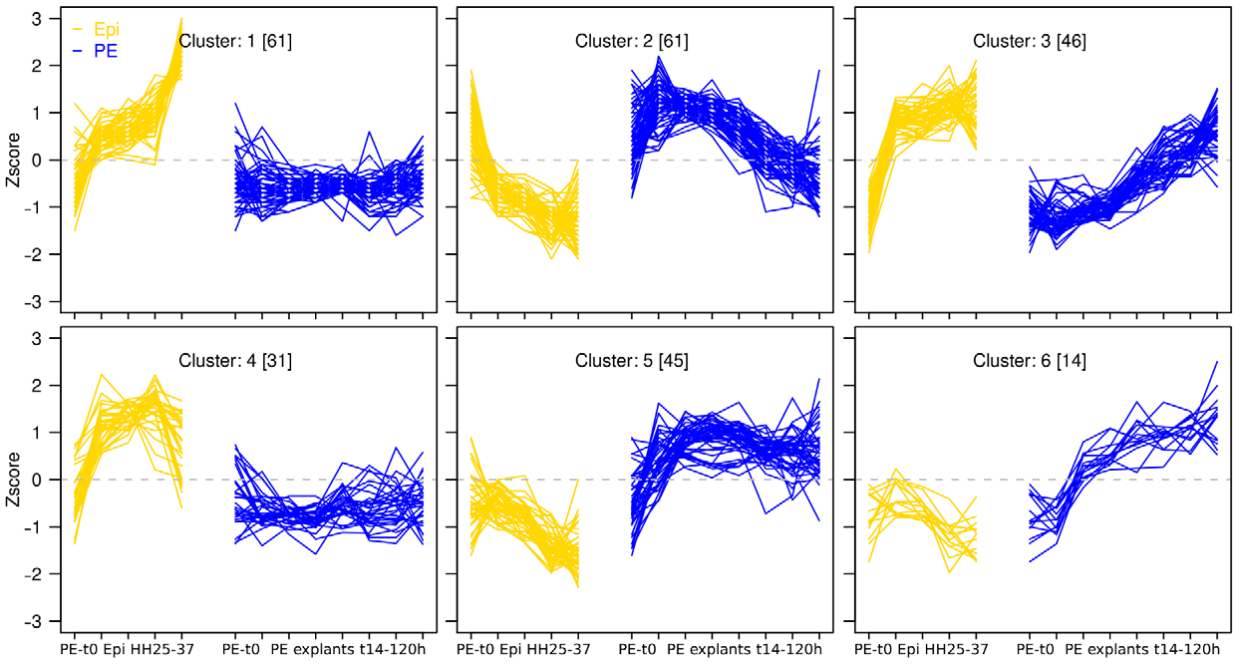
EpiLock. Expression profiles for genes with divergent expression between the Epi and cardiomyogenic lineages. Y-axis represents Zscore-transformed expression levels. Blue and yellow lines represent expression patterns for Epi and PE explant cultures, respectively.

Genes with divergent expression profiles between the PE and Epi differentiation series were considered to be associated with the Epicardial lock. In total 258 genes were identified that showed these divergent expression profiles, and these genes were clustered into 6 discrete expression profiles (Figure 3). Interestingly, for the PE explant data (blue lines), genes in cluster 2 of this combined analysis contains genes with a similar transient expression profile as was observed for the gene cluster associated with the cardiac specification from the PE explant analysis from the previous section (Figure 2; cluster3). Moreover, for these genes, the transient upregulated expression profiles during PE explant differentiation coincides with downregulated expression during Epi differentiation (yellow lines), indicating these to be associated with the Epicardial lock. Concept analyses on all genes in this cluster and on the overlapping subset of genes with Figure 2; cluster 3, showed a prominent association with Wnt signaling. A table with concept analyses for all 6 clusters is available in Table S2.

Although Wnt signaling has repeatedly been shown to be involved at distinct stages of cardiovascular differentiation and disease [2], [19], and was prominently associated with distinct clusters of differentially expressed genes in our analyses, many of the individual Wnt signaling components do not have clearly defined roles in cardiomyocyte differentiation. Upon further inspection of the overlapping genes of these two clusters, the extracellular wnt signaling antagonist Wif1 was selected as a candidate for functional intervention studies in order to define its role during cardiomyocyte differentiation. Moreover, Wif1 is an extracellular acting factor, which makes it an excellent candidate for exogenous manipulation of cellular fate. Therefore, for the remainder of this manuscript we will focus on delineating roles of Wif1 during cardiomyocyte differentiation. qPCR confirmed the differences in expression level for Wif1 (Figure 1 C&D) between the PE and Epi series as well as for several other genes, e.g., Tll1, Spry2, Cyr61 (Figure S2A). Overall, over 90% of all gene-expression profiles could be confirmed by qPCR, although quantitative differences between the methods were observed (this paper & data not shown).

### Wif1 stimulates cardiomyocyte differentiation in PE-explant cultures

**Figure 4:**
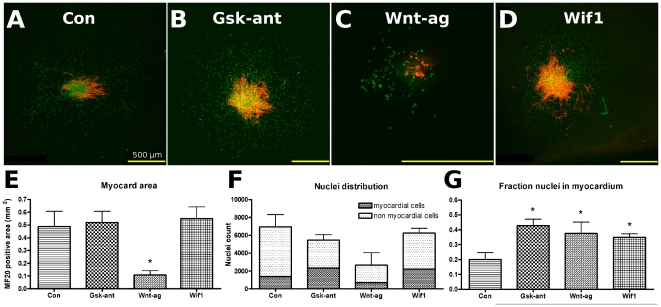
Recombinant protein PE. Immunofluorescent staining for cardiomyocytes (red) and nuclei (green) in control PE-explant cultures (A, n = 5 ) or explant cultures incubated with Gsk3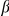 antagonist (B; 5 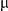
M, n = 7), Wnt-signaling agonist (C; 5 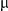
M, n = 6) or Wif1 (D; 50 ng/mL, n = 11). Compounds were added to the cultures in both the collagen gel and M199 medium. Cultures were fixed and analyzed at day 5. The yellow bar at the right bottom represents 500 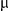
m. Quantified cardiomyocyte area, myocardial vs non myocardial cellular distribution and the fraction of myocardial cells from these explant cultures are plotted in Figure E through G, respectively. Bars represent means 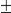 SEM. * indicates a significant difference relative to untreated control conditions.

To establish the role of Wif1 in cardiomyocyte differentiation, PE-explants were cultured for 5 days in the presence of either human recombinant Wif1, pharmacological canonical Wnt-signaling agonist (Cat# 681665, Calbiochem) or Gsk3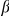 antagonist SB415286. Representative examples of immunofluorescently stained explants are shown in Figure 4A through D. In these cultures the total myocardial cell area, total number of cells and the fraction of cardiomyocyte were quantified. A significant reduction in the myocyte area compared to control explants was observed in cultures treated with Wnt agonist while no significant effects were observed for Gsk3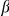 antagonist SB415286 or Wif-1 recombinant protein (Figure 4E). Although the total number of cells was less for all three treatments compared to control (Figure 4F), the fraction of cells within the myocardial area was significantly increased upon treatment with Wif1 and both the Wnt agonist and Gsk3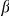 antagonist compared to control (Figure 4G). Taken together, these data indicate that Wif1 stimulates cardiomyocyte differentiation in PE-explant cultures.

### Wif1 exhibits biphasic effects on cardiomyocyte differentiation in p19cl6 cells

In parallel to the analyses in PE-explants, we also performed a series of signal transduction perturbations to investigate the role of Wif1 during first heart field cardiomyogenesis using the DMSO-induced cardiomyocyte differentiation in the mouse pluripotent embryogenic carcinoma cell line p19cl6. Cardiomyocyte differentiation was evident from increased Atp2a2, Gata4 and Myl2 expression (Figure S2B). Expression of Mesp1, an early cardiac mesodermal marker, peaked at 2 days after the onset of differentiation and was maintained at approximately 5-fold higher expression levels relative to control conditions from day 4 onward (Figure S2B). From day 10, spontaneously beating clusters of cells were observed in all DMSO treated cultures (data not shown). Wif1 gene-expression was significantly increased during differentiation albeit with different expression patterns in time than were observed for the chicken PE cultures (Figure 1 C–E).

**Figure 5:**
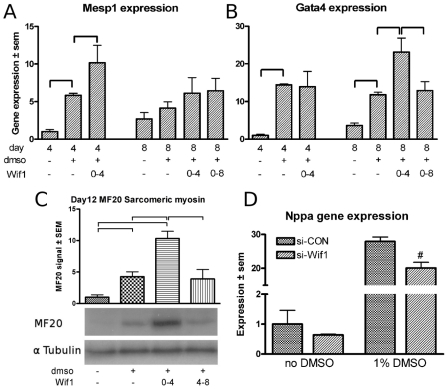
p19cl6 intervention studies. Mesp1 and Gata4 gene expression in p19cl6 cells treated with Wif1 recombinant proteins (A&B). Time of harvest, DMSO exposure and protein exposure time intervals (prot time) are indicated at the x-axis. Bars represent mean expression 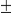 SEM. C: Representative western Blot analysis with MF20 and 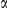
Tubulin at day 12, and quantification of the western blot analysis. Y-axis represents average MF20 to 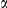
Tubulin ratio 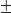 SEM of six independent samples. Day 12 samples without DMSO were set to 1. D: Nppa gene-expression levels following siRNA-mediated knockdown of Wif1 compared to control siRNA conditions. * indicates a significant difference between conditions as indicated in the graphs.

P19cl6 cells were stimulated with recombinant Wif1 at distinct time intervals in the presence or absence of 1% DMSO. Evaluating cardiomyocyte differentiation in these cultures showed that stimulation with Wif1 in the absence of DMSO did not significantly alter the expression level of Gata4 or Mesp1 after 4 or 8 days of culture compared to controls (data not shown). When p19cl6 cells were treated with Wif1 during the first 4 days of the culture in the presence of DMSO, a significant increase in Mesp1 gene expression was found at day 4 of the culture (Figure 5A) and in Gata4 expression at 8 days of culture (Figure 5B). However, when the cultures were stimulated with Wif1 for 8 days in the presence of DMSO the increase in Gata4 expression observed at 4 days was no longer found (Figure 5B). This biphasic effect of Wif1 on the induction of myocyte differentiation was also observed for the protein level of sarcomeric myosin heavy chain protein (Figure 5C). Quantification of myosin heavy chain expression levels after 12 days of culture in the presence of DMSO, showed a 5-fold increase compared to controls. Stimulating these cultures with Wif1 during the first 4 days of culture resulted in an almost 3-fold higher expression level (12-fold relative to control), whereas addition of Wif1 from day 4 until 8 did not result in an attenuation of the expression level of myosin heavy chain.

To further substantiate these observations Wif1 expression was knocked down using gene-specific siRNA. Wif1 knockdown was confirmed at 2 days after transfection (Figure S2C). At 4 days after transfection, Wif1 gene knockdown could still be observed, although at a reduced level (data not shown). The effects of reduced Wif1 levels on cardiomyocyte differentiation were evaluated at four days after transfection. In line with the stimulatory effect of Wif1 protein supplemented to the culture, siRNA mediated Wif1 gene knockdown resulted in a significant reduction of Nppa gene expression in the presence of DMSO (Figure 5D), however, no effects on Mesp1 or Gata4 expression levels were observed (Figure S2C). These relatively mild effects of Wif1 knockdown at the early stages during cardiomyogenesis may be explained by the fact that endogenous Wif1 in p19cl6 cells is upregulated from day 8 onward (Figure 1E).

**Figure 6:**
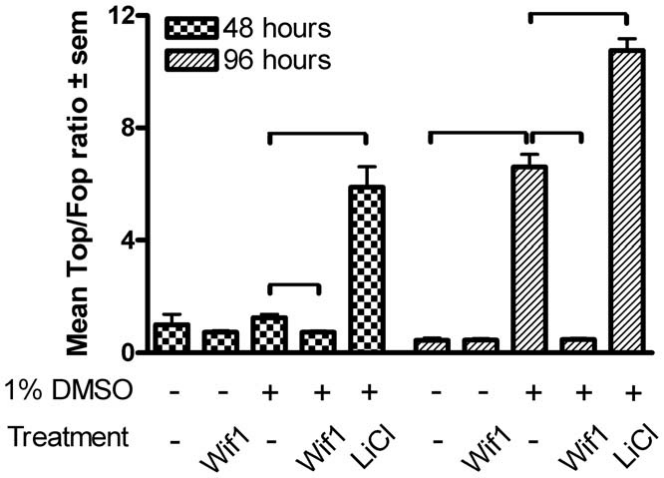
Luciferase assay Wif1. Luciferase assays measuring the Top (4× TCF4 binding sites) to Fop (mutated TCF4 binding sites) ratio as a measure for nuclear activity of endogenous 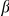-catenin at 48 and 96 hours incubation with the assigned experimental conditions. * indicates a significant difference between conditions as indicated in the graphs.

A previous study using p19cl6 cells [20] has shown that Wnt antagonism and Wnt stimulation operating via the canonical Wnt/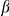-catenin pathway, blocks or augments cardiomyocyte differentiation, respectively. By contrast, our data shows that Wnt inhibition by Wif1 augments differentiation. This opposite effect may be explained by differences in the incubation timing and/or the Wnt signaling modulators used. In order to characterize Wif1 mediated effects on canonical Wnt signaling, we performed a series of 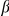-catenin/TCF-responsive Luciferase reporter assays [21] and calculated the Top (4× TCF4 binding sites) to Fop (mutated TCF4 binding sites) ratio as a measure for nuclear activity of endogenous 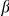-catenin (Figure 6). Incubation of p19cl6 cells with 20 mM LiCl, which induces stabilization and nuclear translocation of 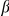-catenin via inhibition of Gsk3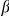, leads to an expected increase in the Top/Fop ratio at both 48 and 96 hours. Although a small but statistically insignificant increase was found after 48 hours of differentiation in the presence of 1% DMSO, 96 hours of incubation resulted in a 14-fold increase in the Top/Fop ratio relative to control conditions. Wif1 incubation for 48 hours in presence of 1% DMSO leads to a significant 42% reduction of the Top/Fop ratio and completely abolished the increase in the Top/Fop ratio at 96 hours.

Taken together, the siRNA transfection and the protein incubation data point to a biphasic effect of Wif1 via 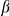-catenin signaling on cardiomyogenesis in which early exposure enhances and late exposure attenuates cardiomyocyte differentiation in p19cl6 cells.

### Wif1 modulates cardiac development in vivo

**Figure 7:**
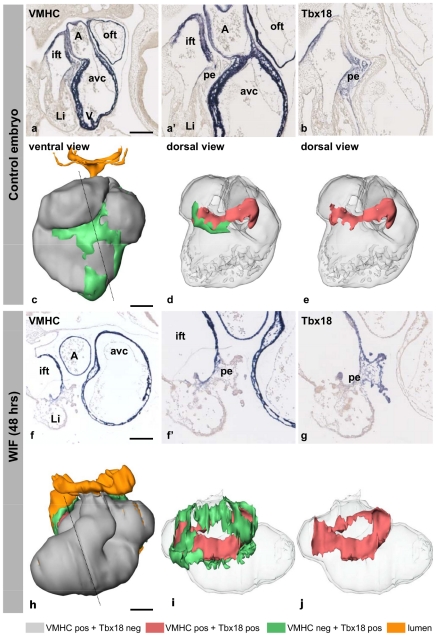
In ovo Wif1. Expression pattern of VMHC and Tbx18 in the hearts of control and Wif1 treated embryos after four days of incubations (stage HH19-20). Wif1 exposure lead to an expansion of the Tbx18 expression domain upstream of the heart. Interestingly, also the Tbx18− and VMHC-positive domain upstream of the heart is much more extensive than observed in controls (B vs G). Moreover, it is evident that in the WIF1 treated embryos no embryonic epicardium is formed resulting in a thinning of the ventricular myocardium (A,A′ vs F,F′). Ventral (C,H) and dorsal, (D,E,I,J) views of 3D cardiac reconstructions indicating VMHC+/Tbx18− (gray), VMHC+/Tbx18+ (red), VMHC−/Tbx18+ (green) and lumen (yellow) compartments. Dashed lines indicate the position of the respective sections. Abbreviations: oft indicates outflow tract; A, atrium; ift, inflow tract; avc, atrioventricular canal; li, liver; V, ventricle; pe, proepicardium.

The results from both the PE-explant cultures and the p19cl6 experiments argue for a prominent role of Wif1 in cardiomyogenesis. In order to confirm these findings in vivo, we treated chicken embryos in ovo from HH12 until HH19-20 with Wif1 recombinant protein. The development of the cardiovascular system and liver was severely impaired (Figure 7 and Figure S3). The ventricular chamber expanded dextro-laterally instead of caudo-ventrally, causing the outflow tract to have a sharp hinge to the right. The three pairs of pharyngeal arch arteries were present and connected to the dorsal aortae. Throughout the heart the myocardium was very thin and small trabeculae were present at the detro-lateral side, indicating that ventricular chamber formation was induced. At the dorsal side of the heart the vessels patterned normally. The PE (Figure 7; green) was normally formed on both the left and right sinus horns. However, at this stage of development the PE villi at the left sinus horn would have disappeared. The bilateral PE villi had expanded and reached the dorsal aspect of the heart, but did not cover the myocardium of the heart as is observed in controls (Figure 7 D,E). Using Tbx18 mRNA expression as a marker for the progenitor population at the inflow of the heart, the Tbx18-expressing domain was much more extensive in Wif1-treated compared to control embryos (Figure 7B,E vs 7G,J). Basically all mesothelium and underlying mesenchyme covering the large veins that flank the pericardial cavity were Tbx18-positive in Wif1-treated embryos. As this Tbx18-positive progenitor pool also contributes to the inflow myocardium, the cardiomyocytes were visualized using a probe to ventricular myosin heavy chain (VMHC, MYH7) mRNA (Figure 7A,A′,F,F′). A large part of the Tbx18-expressing cells upstream of the heart expressed VMHC. The Tbx18− and VMHC-expressing cells were found directly adjacent to the VMHC-positive and Tbx18-negative myocardium of the heart and below the PE; Tbx18 was only expressed in the villous part of the PE. The Tbx18−, VMHC-expressing area was surrounded by a region of Tbx18-positive and VMHC-negative cells. These findings suggest that the Tbx18 progenitor pool upstream of the heart expands and differentiates into cardiomyocytes, but are not integrated into the heart, resulting in a myocardial sleeve covering the inflow vessels.

## Discussion

### Epicardial lock

Cardiomyocytes that are lost during disease are not sufficiently replaced, due to the limited regenerative capacity of the heart. Supplementing additional cardiomyocytes to the heart would be an option to strengthen the heart. However, thus far, approaches supplementing stem cells of different origins have only resulted in slight transient improvement of cardiac function [22]. An alternative approach would be to reprogram epicardial-derived cells that replace the lost cardiomyocytes in such a way that they can differentiate into cardiomyocytes. Although the epicardial-derived cells have the potential to differentiate in another cell type [23], the factors to redirect their differentiation into cardiomyocytes are not known. Because the epicardial-derived cells have been suggested to comprise a stem cell like population [24] and it has previously been shown that part of the proepicardial cells spontaneously differentiate into cardiomyocytes and embryonic epicardial cells do not upon culturing [7]–[9], [25], these cell populations might be a source to identify genes that prevent differentiation of epicardial(-derived) cells into cardiomyocytes, i.e., the epicardial lock.

Recently, claims have been made that an Tbx18-positive epicardial-derived cell population contribution to the myocardial compartment in mice [26], [27]. This, however, has been disputed by others [28] as Tbx18 is expressed early in the myocardium. Nevertheless, no epicardial derived myocardial compartment has been described during chicken cardiogenesis [25], advocating the chicken as a legitimate model system to investigate processes associated with PE -Epi lineage divergence.

By comparing the changes in gene-expression profiles of the different stages of cultured proepicardial cells with the different stages of embryonic epicardial cells, we were able to identify many genes in these two lineages that had divergent profiles and therefore may be associated with the epicardial lock. Of particular interest are genes that, in addition to displaying divergent expression profiles, are also associated with cardiac specification. i.e., that show a transient increase in expression early during PE differentiation towards cardiomyocytes in explant cultures. Our analyses showed that Wnt signaling components were one group of molecules that were prominently present in this subset of genes, in addition to the many other Wnt-related components that our array analysis had identified, like Wnt2a and Wnt5b, Frizzled receptors Fzd1, 2 and 7, Frzb, dickkopf homolog 1, Wnt1 inducible signaling pathway protein-1 and 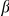-catenin. Specifically, the extracellular Wnt antagonist Wif1, was chosen as a follow-up candidate to delineate its role during cardiomyogenesis in models for the first and second heart fields using the p19cl6 cell line and PE explant cultures, respectively.

### Wif1 functional interventions

Little is known about the role of Wif1 in cardiogenesis. Schneider et al. found that injecting mRNA coding for Wif1 in Xenopus ventral marginal zone explants only weakly induced Nkx2.5 expression [29]. In our p19cl6 intervention studies, limiting Wif1 exposure to the first 4 days during during culture, lead to induced Gata4 expression at day 8 of culture, while the prolonged exposure up to day 8 blocked the increase in Gata4, suggesting an increase in the cardiomyocyte progenitor pool through early exposure. Western blot analysis for sarcomeric myosin on day 12 samples confirmed this biphasic effect at the protein level. Moreover, in vivo Wif1 incubation indicated that the Tbx18 positive cardiac progenitor pool upstream of the heart expands and differentiates into cardiomyocytes precociously.

Studies have shown canonical Wnt signaling to be biphasic in nature in embryonic stem cell like models, i.e., early Wnt activation stimulates while late Wnt activation inhibits cardiomyocyte differentiation [30], [31]. The Gata4 and Mesp1 expression profiles in response to Wif1 in p19cl6 cells would indeed imply an early phase of activated Wnt signaling, followed by a phase of Wnt signaling inhibition. In the chicken PE-explant cultures we observed that both Wnt activation and Wif1 incubation resulted in a significant increase in the fraction of cardiomyocytes after 5 days in culture. This was unexpected since Wif1 was described by Hsieh et al. [32] as an extracellular inhibitor of Wnt signaling through sequestration of Wnt proteins and our Top/Fop Luciferase reporter assays clearly established that Wif1 is able to block 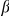-catenin signaling (Figure 6). Thus, in addition to the biphasic nature of the Wnt response, there may also be a dual response to Wif1 exposure. It is possible that this biphasic and dual response relate to the differences in response to canonical and non-canonical Wnt signaling. While early and late Wnt activation stimulates and inhibits cardiomyocyte differentiation, respectively [30], [31], non-canonical Wnt signaling, via Wnt11 has been shown to enhance cardiomyogenic differentiation through PKC and JNK-mediated signaling (reviewed in [33]). In addition, Afouda et al. indicated that the GATA transcription factors reside at a central position in between the Wnt signaling cascades during cardiomyogenesis [34]. Moreover, extensive cross-talk between canonical and non-canonical has been described and it is becoming more evident that the Wnt signaling cascades can no longer be regarded as linear or stand alone signaling entities [35], [36]. Therefore, it could be that Wif1, next to its Wnt sequestration property, stimulates the calcium and/or JNK dependent non-canonical pathways. This could explain Wif1's dual properties by manipulating the balance between canonical and non-canonical Wnt signaling for enhancing cardiomyocyte differentiation.

### General conclusion

In summary, our study provides a thorough description of gene-expression alterations that are associated with PE to cardiomyocyte differentiation. Moreover, we are the first to deliver a detailed description of gene-expression alterations that are associated with the processes that constrain the embryonic, and adult, Epi from differentiating into the cardiomyocyte lineage. The data is publicly available for data mining via the GEO database (GSE13923). Functional genomics will be required to ascertain whether any of these genes could unlock the cardiogenic potential in epicardial(-derived) cells in order to use these cells for cardiac regeneration therapy. Many factors are implicated in establishing the Epi-Lock suggesting broad acting mechanisms underlie the lineage restriction, potentially with Wnt signaling residing at a central position. Still, it is unlikely that any individual factor will be able to fully reinstate the cardiogenic capacity in epicardial(-derived) cells. Identifying and targeting common features of the differentially-expressed genes will be pivotal in these efforts. Additional experiments focusing on DNA methylation, histone modifications and microRNA expression may be necessary to fully appreciate and to break the epicardial lineage restriction. In addition, for one of the factors involved in the Epicardial lock, Wif1, we show with model systems for the first and second heart fields that it enhances cardiomyocyte differentiation in chicken PE explant cultures, increases the Tbx18-positive cardiomyocyte progenitor pool in chicken embryos stimulates cardiomyocyte differentiation in the mouse p19cl6 cell line.

## Materials and Methods

### Chicken embryos, proepicardial and epicardial explant cultures

Fertilized chicken eggs were obtained from a local hatchery (Drost BV, Nieuw Loosdrecht, The Netherlands), incubated at 39

C in a moist atmosphere, and automatically turned every hour. After the appropriate incubation times, embryos were isolated in Earl's balanced salt solution without phenol red (EBBS, MP Biochemicals) and staged according to Hamburger and Hamilton [10]. Collagen gels were prepared and PE were isolated and cultured as previously described [9], [37]. In short, PE from stage HH16-17 embryos were cut at the base to prevent liver primordium or sinus venosus contamination. Up to 20 individual PE were placed on a drained collagen gel and allowed to attach overnight after which complete M199 medium (M199 medium containing penicillin/streptomycin (Life Technologies), 5 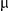
g/mL insulin, 5 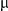
g/mL transferrin and 5 ng/mL selenium (ITS, Collaborative Research Inc.), 2 mM glutamine (Life Technologies), and 1% chicken serum) was added. At 14 hours in culture, prematurely beating explants, indicative of myocardial cell contamination, were removed. Embryonic Epi tissue samples from chicken embryos at stages HH25 (n = 20), HH29 (n = 16), HH32 (n = 20) or HH37 (n = 9) were processed as previously described [9]. In short, the isolated hearts were placed on collagen gels, allowed to attach overnight after which the hearts were removed leaving the formed Epi monolayers on the collagen surface and complete M199 medium was added. Human recombinant growth factor Wif-1 (R&D systems) was added to PE cultures at a concentration of 50 ng/mL in both the collagen gel and M199 medium. Pharmacological Wnt-signaling agonist (Cat# 681665, Calbiochem) [38] or Gsk3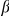 antagonist SB415286 [39] was added at 5 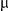
M. PE and Epi-explant cultures were maintained until the indicated time points at which they were either lysed for RNA isolation for array hybridization or real-time PCR analyses or fixed and immunofluorescently stained. Myocytes were visualized using MF20 antibody (Hybridoma bank, Iowa City, IA, USA) and Goat anti Mouse Alexa488 (Molecular Probes) and nuclei using SytoxOrange (Molecular Probes). The total area occupied by cardiomyocytes and the total number of nuclei were determined using an user-written macro in Image Pro-Plus 5.0 (measure_myo_fraction_v02), as previously described [7].

### Wif1 in vivo assay and 3D cardiac reconstructions

After 48h of incubation, the eggs were windowed and if the embryo had developed to stage 12, Wif1 was injected directly in the yolk below the embryo to a final concentration of 50 ng/mL, taking into account the diluent volume of the egg. Control embryos were injected with growth factor solvent. After 24 hours the embryos were again injected with the same amount of Wif1, and re-incubated for another 24 hours (stage 19–20). Upon isolation the embryos were staged, fixed in 4% paraformaldehyde in PBS and embedded in paraplast. Upon sectioning (10 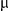
m) the mRNA of Tbx18 [7] and VMHC [40] was visualized using in situ hybridization [41]. As the morphology and patterns of gene expression were similar in all six embryos, one was 3D-reconstructed using AMIRA as described previously [42].

### RNA isolation, amplification and fluorescent labeling

Total RNA for array analyses was isolated from separate pools of PE at stage HH16, PE explants at 14, 24, 36, 48, 60, 72 and 120 hours in culture and from Epi at stages HH25, HH29, HH32 and HH37 at two days in culture. Cells were lysed in excess RA1 buffer containing 1% (v/v) 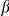-mercaptoethanol and further processed according to the manufacturers protocol including a DNase I treatment (NucleoSpin RNA L, Macherey-Nagel). RNA concentration was determined using a Nanodrop 1000 (Thermo Scientific) and RNA integrity was checked on a Agilent 2100 Bioanalyzer. 500 ng total RNA was amplified using the MessageAmp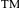 kit (Ambion) with incorporation of 5-(3-aminoallyl)-UTP (aaUTP) and UTP in a ratio of 2∶3 as previously described [43]. For each array channel 1.5 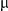
g amplified RNA was labeled with either mono-reactive fluorescent Cy3 or Cy5 dye (Amersham) and stored at −80

C prior to array hybridization.

### Array hybridization & image processing

Chicken oligo arrays representing 20460 long oligonucleotide probe sequences printed in singlets on Corning Epoxide Coated Slides were purchased from ARK-Genomics, Scotland, UK. Slides were pre-hybridized (5× SSC, 25% (v/v) formamide, 0.1% (w/v) SDS, 1% (w/v) bovine serum albumin, fraction V (Sigma)) for 45 minutes at 42

C to block the remaining reactive epoxide groups and reduce background signal. Cy3 and Cy5 labeled RNA samples were combined as described in Figure S1 and mixed with 2× hybridization mixture to a final concentration of 5× SSC, 25% (v/v) formamide, 0.1% (w/v) SDS, 0.2 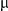
g/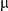
L herring sperm DNA (Invitrogen). Target-RNA mix was denatured for 5 minutes at 70

C and incubated at 42

C for 30 minutes. Hybridization was done in a GeneTAC hybridization station (Genomic Solutions) for 16 hours at 42

C with agitation. The arrays were washed at room temperature in 5 successive buffers, i.e., 1× SSC+0.2% SDS, 0.1× SSC+0.2% SDS, 1× SSC, 0.1× SSC and 0.001× SSC, each step lasting 4 minutes. The slides were finally pressure-air dried and stored in the dark. Slides were scanned using an Agilent G2565BA microarray scanner on a 10 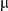
m resolution. The resulting images were split into the red and green channels prior to feature extraction with the GenePix Pro 6.1.0.2 (Axon Instruments Inc.) software. Spots with high local background or aberrant spot shape were flagged by the software and manually checked.

### Array data transformation and statistics

All array data was processed using Bioconductor packages [44] in R-statistics. Median spot intensities were imported from the genepix results files using the Limma package. Flagged spots were assigned a weight of 0.1, control spots were removed and the remaining data were normalized with lowess (parameters: span = 0.25, iterations = 2) and aquantile transformations [45]. The raw microarray data have been deposited in the MIAME compliant GEO database [46] as GSE13923. Significant differences in gene expression for the Epi series were determined by fitting the linear model Epi stage+dye followed by testing all possible pair-wise comparisons between the four time points in Limma [47]. Probes with Benjamini-Hochberg linear step-up false discovery rate (BH-FDR) [48] corrected p-values below 0.01% were considered to be significantly differentially regulated. The PE differentiation time-course series was analysed using a temporal Hotelling T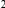-test [12], [13]. A second degree-polynomial curve was fitted through the 16 data points while conserving the temporal order and correcting for the dye effect. A probe was considered to show differential regulation in time when any of the individual parameters of the polynomial, except for the intercept, are significantly different from zero at a BH-FDR of 15%. Significantly-regulated genes were K-means clustered [49] into 7 clusters, each of which was further analysed to explore functional associations between the differentially-expressed genes with Anni 2.0 [15]. Official gene names were retrieved for each probe and matched to their corresponding concept profiles in the Anni program. Probes that display divergent expression profiles between the Epi and PE differentiation series were identified by correlating gene-expression levels to a set of predefined divergent expression profiles. Probes with a statistically significant Pearson correlation (BH-FDR<1%, absolute Pearson coefficient above 0.6) were considered to display significantly divergent expression profiles between the PE and Epi series. Array probe annotations used in the analyses described in this manuscript were a merge of those obtained from the GEISHA resource website [50] and a custom annotation package compiled using R/AnnBuilder package.

### Mouse pluripotent embryogenic carcinoma cell culture

p19cl6 cells expressing Green Fluorescent Protein (GFP) under the transcriptional control of the 250 bp MLC-2v promoter [51], a kind gift from Christine Mummery, were cultured in DMEM∶F12 (1∶1) media (Invitrogen) supplemented with 7.5% fetal calf serum (HyClone), 0.05 mM non-essential amino acids, 100 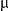
g/mL Streptomycin, 100 U/mL Penicillin and 600 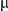
g/mL G418 (Invitrogen). Medium was refreshed every other day. For cardiomyocyte differentiation series, cells were plated at 2000 cells per well in TC treated 6 wells plates (Corning) and allowed to attach overnight. Differentiation was induced by 1% DMSO for four days. Gene specific Silencer select siRNA sequences targeting Wif1 (s76937, s76939) and siRNA Negative Control #2 were obtained from Ambion. Both siRNA sequences per gene were tested on their ability to reduce target expression levels. The best performing one was used in the described experiments. Transfection using Lipofectamine2000 (Invitrogen) was performed according to the manufacturers protocol. Optimal siRNA∶Lipofectamine2000 ratios were determined to be 0.2 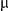
L Lipofectamine2000 per pmol siRNA (data not shown). All experiments were performed at a 10 nM siRNA concentration. Recombinant protein Wif1 was added to the culture medium at a final concentration of 3nM at the indicated time intervals. Differentiation cultures were maintained up to 12 days.

### Quantitative Real-Time PCR

For qPCR measurements complementary DNA strands were generated from 250–500 ng total RNA in a 20 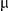
L reaction volume using either Superscript III with an anchored oligo(dT) reverse transcription primer (Invitrogen) or RevertAid H Minus M-MuLV Reverse Transcriptase with random hexamer primer (Fermentas) for PE and p19cl6 samples, respectively. An equivalent of 1 ng total RNA was used in the PCR amplification with 100 nM gene-specific primers and 4 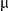
L iQ SYBR Green Supermix (bioRad) in an 8 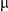
L reaction using standard cycle parameters on an LightCycler480 (Roche). Gene-specific primers (Invitrogen) were designed using Primer Express v2.0 (Applied Biosystems) to be in close proximity to the array probe sequence, to span exon-exon junctions and to have an annealing temperature of 60

C and with an amplicon Tm range between 80 and 85

C. Sequences are available upon request. Threshold values were determined using the 2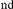 Derivative MaxFactor method in the LightCycler480 software package.

### Western-blot

Cells were washed in cold HBSS and lysed in RIPA buffer (25 mM Tris-HCl pH 7.6, 150 mM NaCl, 1% NP-40, 1% sodium deoxycholate, 0.1% SDS). Equal amounts of protein (BCA kit, Thermo scientific) were separated on a 5% SDS-PAGE gel and transferred to polyvinylidene-fluoride membranes. Protein transfer was confirmed by Ponceau staining. Membranes were blocked o/n with 5% non-fat milk in TBS-T at room temperature and were probed with MF20 (Hybridoma bank, Iowa City, IA, USA, 1/200 dilution) to stain sarcomeric myosin and 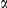
Tubulin (Clone DM1A, Sigma, 1/10.000 dilution) as a loading control. Antibodies were incubated in TBS-T containing 5% nonfat milk for 1 hour at room temperature. Membranes were washed with TBS-T, incubated with the appropriate secondary antibody conjugated to horseradish peroxidase for 30 minutes at room temperature, washed 2× in TBST and 1× in TBS before the bands were visualized by enhanced chemiluminescence (Amersham Biosciences).

### Luciferase assay

p19cl6 cells were seeded at 40k per well in 12 wells plates and allowed to attach overnight. Per well, 25 ng Renilla with 500 ng TOP or FOP [21] expression plasmids were co-transfected using 1.5 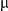
L Lipofectamine2000 in Optimem for 6 hours. After transfection, medium was added to the wells regain standard culture conditions. Cells were treated with 3nM Wif1 recombinant protein or 20 mM LiCl with and without 1% DMSO. Cells were washed with HBSS and lysed in passive lysis buffer (Dual luciferase system, Promega) at 48 and 96 hours incubation. Firefly Luciferase activity (TOP and FOP) was normalized to Renilla luciferase activity per well (Dual-Glo Luciferase Assay system, Promega). Four biological replicates per condition per time point were used in these assays and Luciferase and Renilla activity were measured in triplicate per sample.

### Statistics for real-time PCR, immunohistochemistry and luciferase assays

Differences between experiment conditions for the p19cl6 studies were tested using a students t-test with Bonferroni multiple testing corrections. Cardiomyocyte area and cells count in PE explants were compared using a Kruskal-Wallis nonparametric Anova. Differences with a p-value smaller than 0.05 were considered significant.

## Supporting Information

Figure S1 **Experiment design.** Experiment design for the chicken oligonucleotide microarrays. The 8 PE-explant differentiation samples were hybridized in a 2-color looped experiment design, i.e., hybridization of successive time-points per array, with dye swaps, resulting in four technical replicates for each time point. The four Epi samples were hybridized in all possible pair-wise combinations, with dye-swaps, leading to 6 replicates per time point. To allow for valid comparisons between the Epi and PE differentiation, the two array series were connected via hybridization of both Epi stage HH25 and HH29 with the PE explant at 48 hours in culture, with dye swaps. Each double edged arrow represents two dye-swapped hybridizations. In total 32 arrays were used in this study.
(TIF)

Figure S2
**qPCR data.** A: Confirmation of microarray gene-expression profiles for Tll1, Spry2 and Cyr61 with qPCR. Y-axis represents log2-transformed mean expression levels. Stage HH16 PE expression was set to 0. PE-explant cultures at 24, 48, 72 and 120 hours were compared with all four Epi stages. Ndufb3 was used as an internal control to normalize qPCR gene-expression levels. B: Gene-expression profiles for Mesp1, Gata4, Myl2 and Atp2a2 during p19cl6 differentiation towards a cardiomyocyte phenotype. Hypoxanthine-guanine phosphoribosyltransferase (Hprt) was used as an internal control to normalize qPCR expression levels. Lines represent mean expression levels 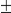 SEM calculated relative to time matched controls. * indicates a significant difference in expression relative to control conditions.C: Evaluation of siRNA mediated knockdown of gene expression at day 2 after transfection for the two individual siRNA sequences for Wif1, i.e.,s76937, and s76939. Hprt was used as an internal control to normalize qPCR gene-expression levels. Bars represent mean gene-expression levels 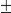 SEM. Expression levels were calculated relative to siRNA Negative Control #2 without DMSO. The best performing siRNA sequence was used in the described experiments, i.e., s76937.(TIF)

Figure S3 **Interactive 3D cardiac reconstruction.** Interactive 3D reconstructions of control and Wif1 treated hearts. In both reconstruction the myocardium (gray), the Tbx18-positive myocardium (red), the Tbx18 positive non-myocardium (green) and the cardiac lumen (yellow) is shown. At the left side the control panel allows to change each structure to be made transparent or to be removed. For convenience four informative preset views have been prepared that can be selected by pushing the respective button. Settings for proper handling of 3D interactive PDF files is Acrobat Reader 9.x can be found under the button marked with a question mark.
(TIF)

Table S1 **Differentially expressed genes.** Differentially-expressed genes for all analyses. Values represent log2 expression levels. The Kmeans clusters to which a gene belongs are indicated in the last two columns.
(XLS)

Table S2 **Concepts Summary.** Concept analysis summary tables for the individual gene lists described. The analyses on the PE Kmean clustering (PE_c1-7), PE-Epi divergence (div_c1-6) and the overlap between PE_c3 and div_c2 (overlap_cPE_c3_div_c2) are described in separate Tab-sheets. Genes with overlapping concept profiles are grouped in sub-clusters indicated in the sc-N format in column1. Individual genes present in the specific list are indicated by gene symbol and their associated concept-identifiers in columns B and C, respectively. Column E describes the top concepts that underlie the clustering of the genes in column B, with their associated concept-identifiers (column F) and the individual contributions (column G) to the clustering. Only concepts with a contribution above 1% are listed.
(XLS)
