# Supplementary figures and images for: Comprehensive Gene-Expression Survey Identifies Wif1 as a Modulator of Cardiomyocyte Differentiation
Source: PLoS One. 2010 Dec 13;5(12):e15504. doi: 10.1371/journal.pone.0015504 (PMC3001492; doi:10.1371/journal.pone.0015504)

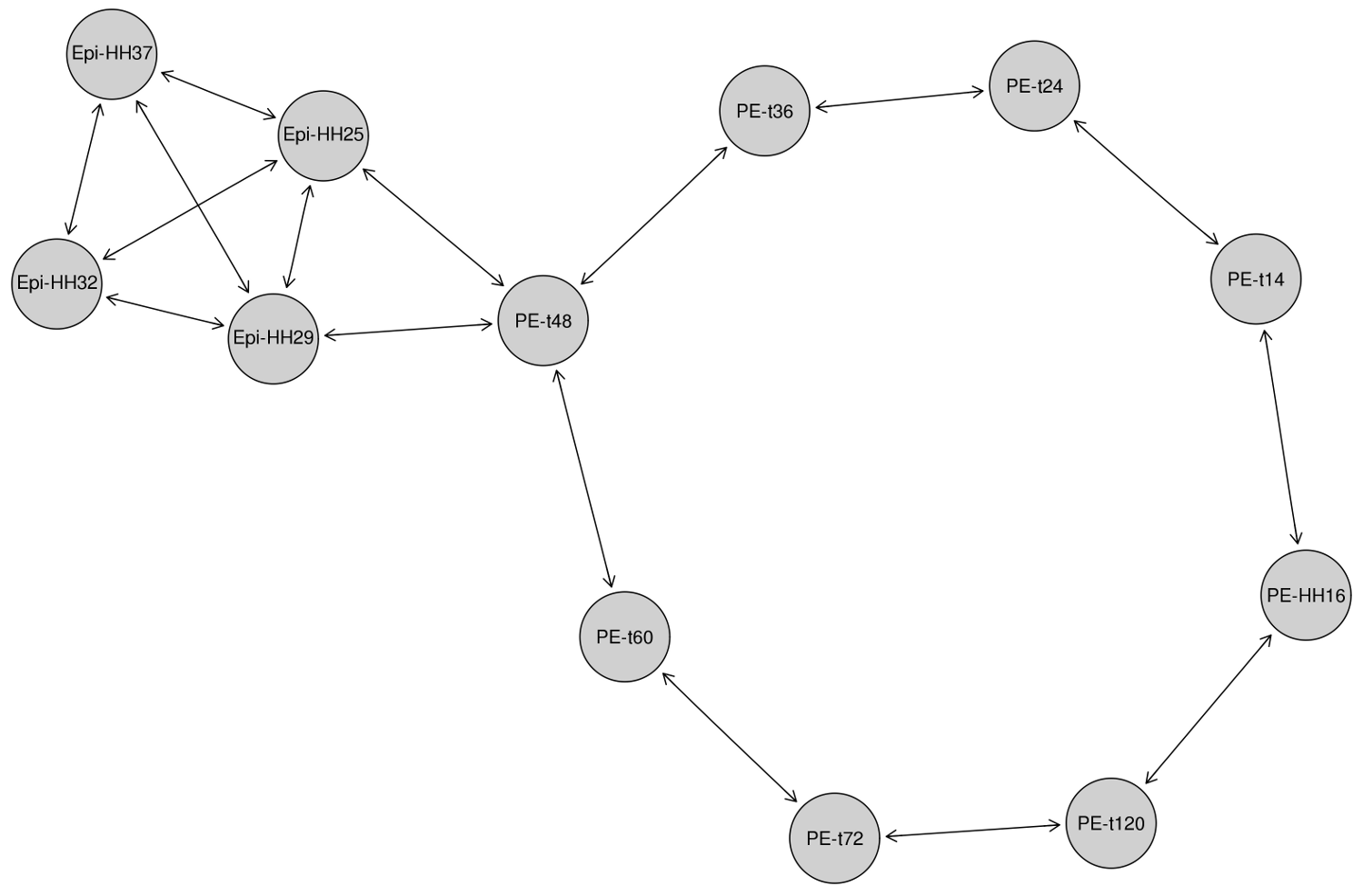

Supplement: Figure S1 — Experiment design. Experiment design for the chicken oligonucleotide microarrays. The 8 PE-explant differentiation samples were hybridized in a 2-color looped experiment design, i.e., hybridization of successive time-points per array, with dye swaps, resulting in four technical replicates for each time point. The four Epi samples were hybridized in all possible pair-wise combinations, with dye-swaps, leading to 6 replicates per time point. To allow for valid comparisons between the Epi and PE differentiation, the two array series were connected via hybridization of both Epi stage HH25 and HH29 with the PE explant at 48 hours in culture, with dye swaps. Each double edged arrow represents two dye-swapped hybridizations. In total 32 arrays were used in this study. (TIF) [file pone.0015504.s001.tif]

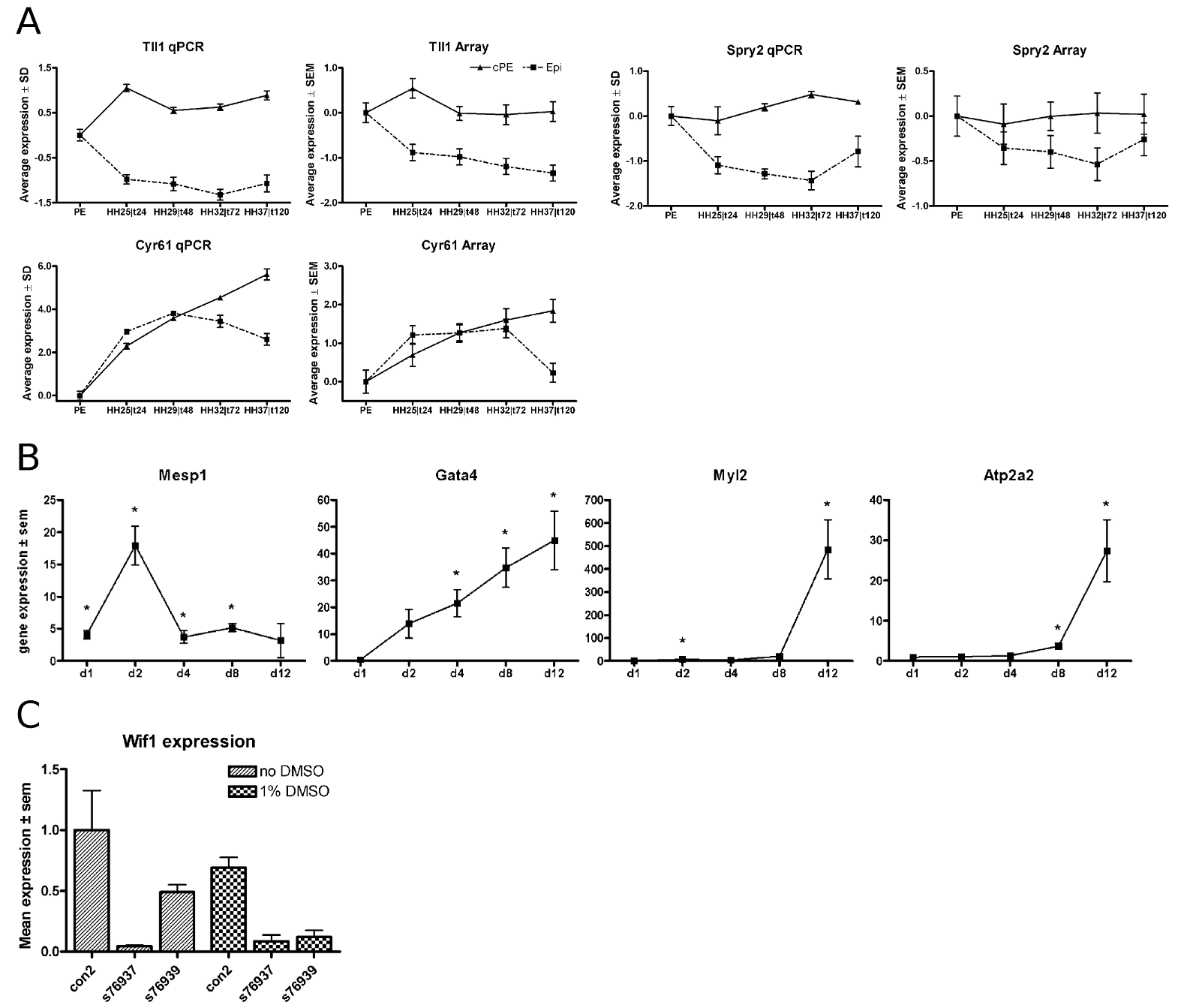

Supplement: Figure S2 — qPCR data. A: Confirmation of microarray gene-expression profiles for Tll1, Spry2 and Cyr61 with qPCR. Y-axis represents log2-transformed mean expression levels. Stage HH16 PE expression was set to 0. PE-explant cultures at 24, 48, 72 and 120 hours were compared with all four Epi stages. Ndufb3 was used as an internal control to normalize qPCR gene-expression levels. B: Gene-expression profiles for Mesp1, Gata4, Myl2 and Atp2a2 during p19cl6 differentiation towards a cardiomyocyte phenotype. Hypoxanthine-guanine phosphoribosyltransferase (Hprt) was used as an internal control to normalize qPCR expression levels. Lines represent mean expression levels SEM calculated relative to time matched controls. * indicates a significant difference in expression relative to control conditions.C: Evaluation of siRNA mediated knockdown of gene expression at day 2 after transfection for the two individual siRNA sequences for Wif1, i.e.,s76937, and s76939. Hprt was used as an internal control to normalize qPCR gene-expression levels. Bars represent mean gene-expression levels SEM. Expression levels were calculated relative to siRNA Negative Control #2 without DMSO. The best performing siRNA sequence was used in the described experiments, i.e., s76937. (TIF) [file pone.0015504.s002.tif]
