# Supplementary material for: Comprehensive Gene-Expression Survey Identifies Wif1 as a Modulator of Cardiomyocyte Differentiation
Source: PLoS One. 2010 Dec 13;5(12):e15504. doi: 10.1371/journal.pone.0015504 (PMC3001492; doi:10.1371/journal.pone.0015504)

Stage 21 Chicken Embryo

structures

- Myocardium
- Tbx18+ Myo
- Tbx18+ NonMyo
- Lumen

preset views

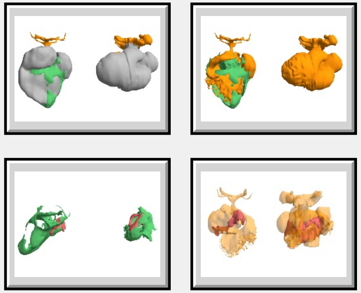

credits

Buermans *et al*
2010

heart failure
research center

Control | WIF1

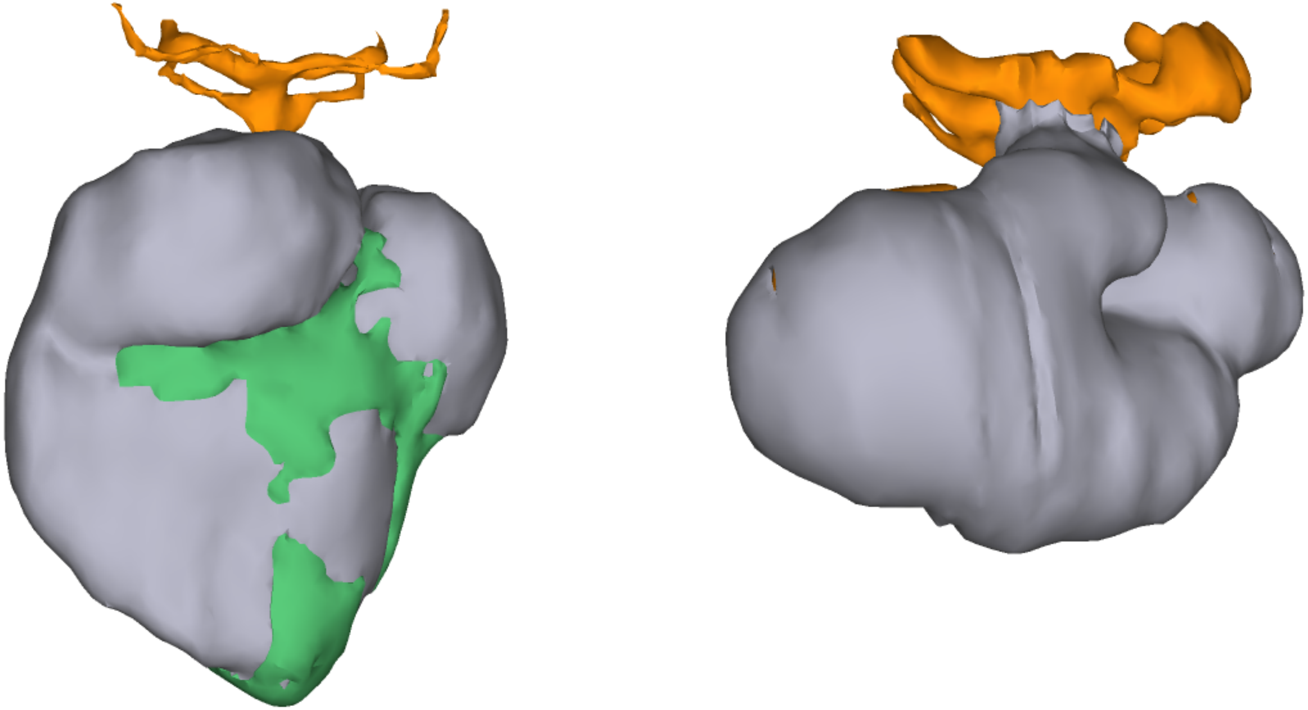

Supplement: Figure S3 — Interactive 3D cardiac reconstruction. Interactive 3D reconstructions of control and Wif1 treated hearts. In both reconstruction the myocardium (gray), the Tbx18-positive myocardium (red), the Tbx18 positive non-myocardium (green) and the cardiac lumen (yellow) is shown. At the left side the control panel allows to change each structure to be made transparent or to be removed. For convenience four informative preset views have been prepared that can be selected by pushing the respective button. Settings for proper handling of 3D interactive PDF files is Acrobat Reader 9.x can be found under the button marked with a question mark. (TIF) [file pone.0015504.s003.tif]
